# Cell‐free DNA profiling in retinoblastoma patients with advanced intraocular disease: An MSKCC experience

**DOI:** 10.1002/cam4.3144

**Published:** 2020-07-07

**Authors:** Prachi Kothari, Francesco Marass, Julie L. Yang, Caitlin M. Stewart, Dennis Stephens, Juber Patel, Maysun Hasan, Xiaohong Jing, Fanli Meng, Jeanette Enriquez, Kety Huberman, Agnes Viale, Jasmine H. Francis, Michael F. Berger, Neerav Shukla, David H. Abramson, Ira J. Dunkel, Dana W.Y. Tsui

**Affiliations:** ^1^ Memorial Sloan Kettering Cancer Center New York NY USA; ^2^ ETH Zurich Basel Switzerland; ^3^ SIB Swiss Institute of Bioinformatics Basel Switzerland; ^4^ Weill Cornell Medical College NY USA

**Keywords:** liquid biopsy, molecular profiling in retinoblastoma, plasma cell‐free DNA, *RB1* mutation, retinoblastoma

## Abstract

**Purpose:**

The enucleation rate for retinoblastoma has dropped from over 95% to under 10% in the past 10 years as a result of improvements in therapy. This reduces access to tumor tissue for molecular profiling, especially in unilateral retinoblastoma, and hinders the confirmation of somatic *RB1* mutations necessary for genetic counseling. Plasma cell‐free DNA (cfDNA) has provided a platform for noninvasive molecular profiling in cancer, but its applicability in low tumor burden retinoblastoma has not been shown. We analyzed cfDNA collected from 10 patients with available tumor tissue to determine whether sufficient tumorderived cfDNA is shed in plasma from retinoblastoma tumors to enable noninvasive *RB1* mutation detection.

**Methods:**

Tumor tissue was collected from eye enucleations in 10 patients diagnosed with advanced intra‐ocular unilateral retinoblastoma, three of which went on to develop metastatic disease. Tumor *RB1* mutation status was determined using an FDA‐cleared tumor sequencing assay, MSK‐IMPACT. Plasma samples were collected before eye enucleation and analyzed with a customized panel targeting all exons of *RB1*.

**Results:**

Tumor‐guided genotyping detected 10 of the 13 expected somatic *RB1* mutations in plasma cfDNA in 8 of 10 patients (average variant allele frequency 3.78%). Without referring to *RB1* status in the tumor, de novo mutation calling identified 7 of the 13 expected RB1 mutations (in 6 of 10 patients) with high confidence.

**Conclusion:**

Plasma cfDNA can detect somatic *RB1* mutations in patients with unilateral retinoblastoma. Since intraocular biopsies are avoided in these patients because of concern about spreading tumor, cfDNA can potentially offer a noninvasive platform to guide clinical decisions about treatment, follow‐up schemes, and risk of metastasis.

## Graphical abstract

The improved treatment strategy for retinoblastoma has led to increased ocular survival and less availability of tissue for molecular profiling. This study demonstrates that the identification of *RB1* mutations by plasma cell‐free DNA and its absence in the buffy coat can conclusively determine whether or not the patient has a somatic *RB1* mutation driving the disease.

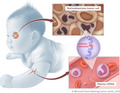

## 1 INTRODUCTION

Retinoblastoma is the most common primary intraocular malignancy of childhood. Two thirds of all cases occur in children less than 2 years old with an age‐adjusted annual incidence in children aged 0‐4 of 10‐14 cases per 1 million.^1^ This corresponds to 1 case per 15,000‐20,000 live births worldwide. About 70% of cases present with unilateral disease.^2^, ^3^, ^4^ The inciting factor leading to development of retinoblastoma is a loss of function mutation in the *RB1* gene on chromosome 13, the first described tumor suppressor.^5^, ^6^ Biallelic mutations of the *RB1* tumor suppressor gene are seen in both heritable and non‐heritable forms of retinoblastoma.^2^, ^7^ In heritable retinoblastoma cases, the initial hit to the *RB1* gene occurs at the germline level and the second mutation occurs in a retinal cell at the somatic level leading to tumor formation. These patients typically present with bilateral disease or multifocal disease earlier in life. In non‐heritable cases, patients develop unilateral tumors. In these cases, two random somatic hits must occur in the *RB1* gene of a single retinal cell to allow for the tumor to arise.^7^, ^8^, ^9^ A small subset of patients who develop unilateral retinoblastoma have been found to have *MYCN* amplification and no *RB1* alterations.^9^, ^10^

Identification and classification of the *RB1* mutation is important for clinical decision making in treatment of retinoblastoma and providing guidance to the patients and their families. Historically definitive standard of care treatment for unilateral retinoblastoma has been enucleation^2^ leading to loss of vision and cosmetic changes; however improvements in treatment options, such as the introduction of ophthalmic artery chemosurgery, has significantly improved ocular survival in patients with unilateral and bilateral retinoblastoma.^11^, ^12^ At Memorial Sloan Kettering Cancer Center, around 95% of patients with retinoblastoma are treated with intra‐arterial chemotherapy or laser to preserve their vision.^11^, ^13^ Furthermore, tumor biopsy at diagnosis is not recommended due to fear of tumor seeding and spread of disease.^14^, ^15^ These factors have led to a reduction in tumor‐based genetic profiling on a large proportion of patients. If tumor tissue is not obtained; a molecular diagnosis cannot be made to determine if the evolution of the patient's tumor derived from somatic mutations only and this hinders definitive clinical *RB1* genetic testing and counseling.

A small percentage of patients with unilateral disease (10%‐15%) carry a germline *RB1* mutation.^2^, ^9^, ^16^ The diagnosis of heritable retinoblastoma can be made clinically in patients who present with bilateral disease or have a positive family history, but a clinical issue arises when a patient presents with unilateral disease, does not undergo enucleation, and a germline *RB1* mutation is not identified in normal blood. While this likely indicates that a germline mutation is not present, the test is formally considered non‐informative since the tumor's *RB1* mutation is unknown and the absence cannot be confirmed in the blood. Therefore, these patients frequently undergo serial ophthalmology exams under anesthesia to assess for development of bilateral disease. The patients with heritable disease are also at higher risk for the development of secondary malignancies and will require additional screening tests as well as additional screening for their families.^17^, ^18^, ^19^, ^20^ With the ability to show that a somatic *RB1* mutation is driving the malignancy, the number of eye exams under anesthesia may be reduced. The patient and family can be reassured that the individual does not have an increased risk of other cancers versus the general population and, therefore, does not need increased cancer screening tests. In addition, the knowledge of a somatic *RB1* mutation can eliminate anxiety concerning the risk of retinoblastoma for current and future family members. Previous reports have shown that DNA from aqueous humor or cerebrospinal fluid in patients with retinoblastoma can reveal tumor‐derived genetic information^14^, ^21^, ^22^; however, obtaining these fluids are still invasive procedures. Hence, a blood‐based noninvasive test for molecular profiling is needed.

**Figure 1:**
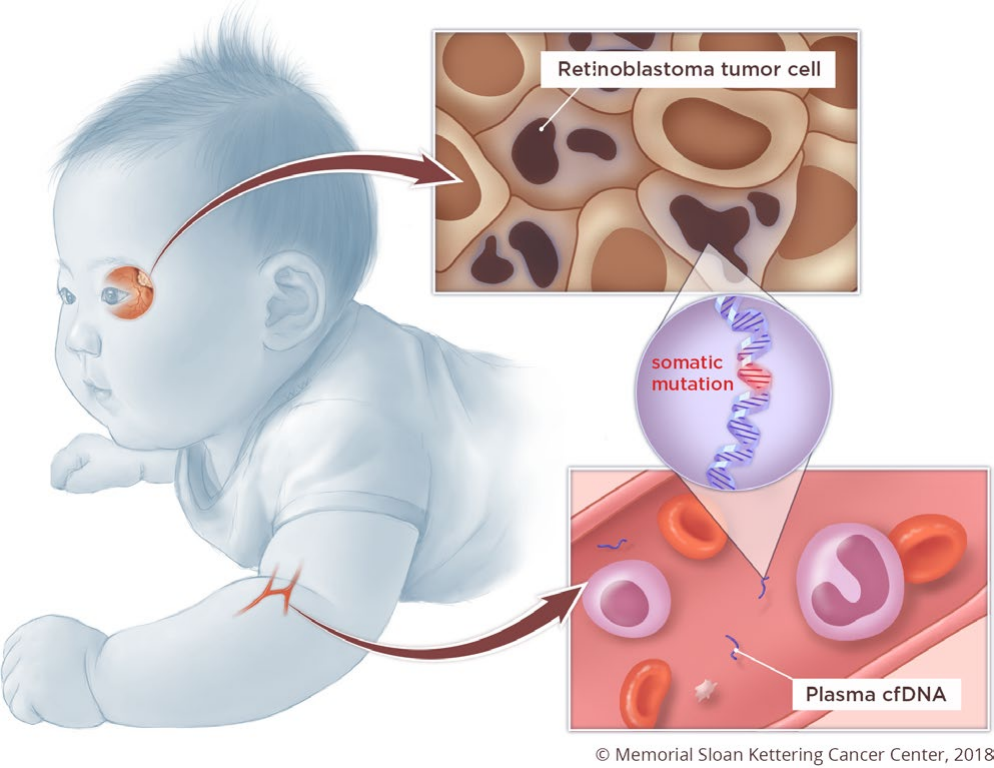
Concept figure showing the application of plasma cfDNA to detect somatic mutations derived from retinoblastoma tumor cells for noninvasive genetic profiling. Reprinted with permission from Memorial Sloan Kettering Cancer Center

Circulating plasma cell‐free DNA (cfDNA) based assays have demonstrated promise as a noninvasive tool for molecular profiling across a broad spectrum of cancer types, aiding in diagnosis,^23^ detecting minimal residual disease, monitoring responses, revealing resistance mechanisms, and tracking clonal evolution during therapy.^24^, ^25^ In plasma, only a small fraction of cfDNA is tumor derived, depending on cancer type, disease stage ^26^, ^27^ and tumor volume.^28^ This tumor‐derived cfDNA is shed into circulation from tumor cells possibly via secretion, apoptosis, and necrosis.^23^ It was unclear whether *RB1* mutations could be detected in cfDNA due to the low burden of disease when compared to other solid tumors. In this proof of concept study, we demonstrate the feasibility of detecting somatic *RB1* mutations in plasma cfDNA of patients with retinoblastoma (Figure 1). We focused on treatment‐naive patients that were planned to receive primary enucleation, in order to minimize any confounding factors introduced by prior treatment.

## 2 METHODS

### 2.1 Patient recruitment and blood collection

This study involved ten patients with advanced intra‐ocular unilateral retinoblastoma who were planned for primary enucleations (Table 1). Three patients from this cohort went on to develop metastatic disease. The table notes each patient's clinical status as of March 2020 as well as specific features of their advanced unilateral disease. This rate of metastasis is relatively higher than the general retinoblastoma population because over 95% of the retinoblastoma patients at MSKCC receive treatment that preserves the eyes, and those who require enucleation tend to have more advanced disease. P19 presented to this institution after receiving 1 cycle of chemotherapy at an outside institution as the parents did not initially consider enucleation for personal reasons. As all of these patients were undergoing eye enucleation, the tumor molecular profiles were used to guide identification of the RB1 mutations in plasma cfDNA.

**Table 1:** Staging at diagnosis and clinical course for this patient cohort

Sample ID	Diagnosis at time of plasma collection	Left eye ICRB (COG)	Right eye ICRB (COG)	Optic Nerve involvement	Choroid involvement	Anterior Chamber involvement	Extra‐ocular extension	Retinal Detachment	Increased intra‐ocular pressure	Buphthalmia	Iris neovascularization	Treatment prior to plasma collection	Development of bilateral disease^a^	Development of metastatic disease^a^	RB1 Clinical Germline Testing
P01	Unilateral	E	N/A	No	No	No	No	Yes	No	No	Yes	No	Yes	No	Heterozygous *RB1* c.1072C > T in exon 11
P23	Unilateral	N/A	E	No	Yes	No	No	Yes	No	No	Yes	No	No	Yes	NA
P11	Unilateral	N/A	E	Yes	Yes	No	No	Yes	No	No	Yes	No	No	Yes	Mosaic del24‐26
P03	Unilateral	E	N/A	No	No	No	No	Yes	No	No	No	No	No	No	Negative
P21	Unilateral	E	N/A	No	No	Yes	No	No	No	No	No	No	No	No	Negative
P22	Unilateral	E	N/A	No	No	No	No	Yes	No	No	Yes	No	No	No	Negative
P26	Unilateral	N/A	E	No	Yes	No	No	Yes	Yes	No	Yes	No	No	No	Negative
P24	Unilateral	N/A	E	No	No	Yes	No	No	No	No	No	No	No	No	NA
P16	Unilateral	E	N/A	Yes	No	No	No	Yes	Yes	No	Yes	No	No	Yes	Negative
P19	Unilateral	N/A	E	Yes	Yes	Yes	No	Yes	Yes	No	Yes	Yes	No	No	NA

Abbreviations: COG, Children's Oncology Group; ICRB, International classification of retinoblastoma.

^a^ Clinical status of patients in cohort through March 2020.

Parents or legal guardians of all the participants in this study provided consent for plasma collection and tumor profiling on an institutional review board approved protocol. A 10mL whole blood sample was collected from each patient in a Streck cfDNA blood collection tube (STRECK). Blood samples were centrifuged with a double centrifugation protocol (1600 g for 10 minutes followed by 14000 g for 10 minutes), and the plasma and buffy coat (portion of blood containing the white blood cells) fractions were separated and stored. Genomic DNA was extracted from the buffy coat using the QIAGEN DNA extraction kit (QIAGEN). CfDNA was extracted from plasma using the QIAGEN QIAsymphony platform (QIAGEN). Quality and quantity of cfDNA were evaluated with automated electrophoresis using Fragment Analyzer with High Sensitivity Genomic DNA Analysis Kit (Advanced Analytical).

### 2.2 Genetic analysis of tumor by MSK‐IMPACT

Tumor molecular profiling from enucleation specimens and patient matched normal blood samples were analyzed by MSK‐IMPACT, an FDA‐cleared clinical targeted sequencing assay that interrogates over 400 cancer‐associated genes as previously described.^29^, ^30^ This panel includes all coding exons in *RB1*. The somatic mutations in *RB1* were identified using a previously described bioinformatics pipeline ^29^, ^30^ and were manually reviewed using Integrated Genomics Viewer (IGV) software version 2.3.36.^31^

### 2.3 Custom targeted sequencing of cfDNA

Plasma cfDNA was sequenced following hybridized capture using a targeted custom capture panel that includes all of the exons in the *RB1* gene. CfDNA libraries were prepared using the KAPA Hyper protocol (Kapa Biosystems). Custom DNA probes targeting the 27 exons of *RB1* and selected regions with heterozygous SNPs were combined in a single capture panel (Integrated DNA Technologies). Pre‐capture libraries were quantified with Qubit (Invitrogen) and an equal mass of each DNA library (~200 ng per sample) was pooled for hybridization capture using a “double capture” protocol modified from the NimbleGen SeqCap Target Enrichment System (Roche). The first capture was incubated at 55°C for 16 hours, followed by post capture purification and 16 cycles of PCR amplification. After PCR clean‐up, the captured target library was processed by a secondary capture incubated at 65°C for 4 hours and followed by post capture purification and 3‐5 cycles of PCR amplification. The resulting pooled, purified libraries were sequenced on the Illumina HiSeq system with 2 × 100 bp paired end reads. The mutation data are available in the cBioPortal for Cancer Genomics at the following URL: https://www.cbioportal.org/study/summary?id=rbl_cfdna_msk_2020.

### 2.4 Data analysis

The detection of somatic mutations from tumor‐derived cfDNA can be performed using two approaches: tumor‐guided genotyping with prior knowledge from the matched tumor, or de novo identification of mutations. Genotyping for a known variant will offer more confidence at lower variant allele frequencies (VAF), as the probability of detecting a false positive in cfDNA that is also present in the matched tumor is very low. De novo identification of mutations requires more stringent criteria to allow for confident calls and to eliminate false positive detection.

To evaluate feasibility of detecting *RB1* mutations in plasma cfDNA, we first performed tumor‐guided genotyping to search for the *RB1* mutations known from the tumor in the corresponding cfDNA samples using Waltz 2.0.^32^ To ensure confidence in the calls made by genotyping in the cfDNA samples, each individual patient's *RB1* mutations were also genotyped in the buffy coat samples of the other patients in the cohort. The average variant allele frequency (VAF) for each specific mutation was calculated in the 9 unmatched buffy coats (Table S1). As the mutations are not expected to be found in the unmatched patients, this calculation serves as a rough indicator of the chance of detecting a false positive for the given *RB1* mutation (possibly arising from errors generated during the data generation process). For genotyping known variants, we considered a variant positive if it fulfills all of the following criteria: (a) have at least 3 supporting reads for the alternative allele; (b) the VAF in cfDNA is higher than the VAF in the matching buffy coat from the same patient; and (c) VAF in cfDNA is greater than the average VAF plus 2 standard deviations across the buffy coats of other patients in the cohort. Any calls that had greater than 50% of reads noted as artifact were noted as not detected.

In clinical practice, tumor tissue is not always available, so to further demonstrate the feasibility of detecting *RB1* mutations in plasma without prior molecular profiles from the tumor, we also attempted to identify *RB1* mutations de novo. The cfDNA samples were analyzed with a mutation calling algorithm called VarDict,^33^ and a more stringent set of criteria was applied to define mutations as positive, with the intent to decrease the chance of reporting false positive mutations. Identified mutations were then filtered with the following criteria: (a) each mutation in plasma cfDNA must have a VAF of >0.5% with at least 10 mutant allele reads; (b) the VAF of a called mutation must be at least 2 times higher than that the VAF found in its matched buffy coat; (c) the VAF of a called mutation in a given patient must be greater than the average VAF plus 2 standard deviations across the 9 unmatched buffy coats. All the variants that passed these filters were manually reviewed on IGV to remove technical artifacts.

## 3 RESULTS

We analyzed plasma cfDNA and tumor tissue from 10 pediatric patients with advanced intra‐ocular unilateral retinoblastoma who all underwent eye enucleation (Table 1). Tumor‐specific somatic *RB1* mutations were identified by MSK‐IMPACT from enucleation samples of all 10 patients. The median age was 30.8 months old at the time of diagnosis (range 0.67‐50.2 months). Nine patients did not receive any treatment prior to plasma collection and eye enucleation. One patient received a cycle of systemic chemotherapy consisting of carboplatin, etoposide, and vincristine prior to plasma collection at an outside institution prior to presentation to this institution Total plasma cfDNA yields ranged from 5.5 ng to 27.4 ng (mean 17.3 ng). The cfDNA samples were sequenced to an average unique coverage of ~1530x.

**Figure 2:**
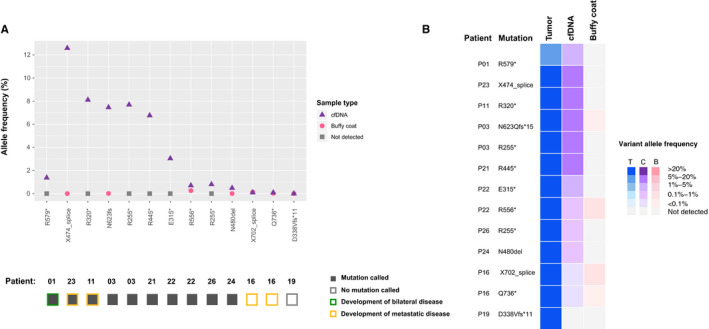
*RB1* mutations detected by tumor directed genotyping. Figure 2A: Variant allele frequencies (VAF) of each *RB1* mutation detected in the 10 plasma cfDNA samples obtained. The solid boxes below are plasma cfDNA samples in which the *RB1* mutation was detected by genotyping. Figure 2B: VAFs compared from tumor, cfDNA, and buffy coats of all 10 patients. AF, allele frequency; T, tumor; cf, cfDNA; BC, Buffy Coat

Based on the *RB1* mutations reported in tumor sequencing, we first attempted to genotype those mutations in the corresponding cfDNA using the criteria outlined in the Methods. We detected somatic *RB1* mutations in 8 of 10 cfDNA samples, 10 of 13 *RB1* mutations (median VAF 4.9%, range 0.7%‐12.6%) (Figure 2). The buffy coat from each patient was concurrently evaluated using the same procedure to filter out germline variants. Of the remaining 2 of 10 cfDNA samples, we observed evidence of tumor guided *RB1* mutations below the detection threshold as defined in the methods in P16 (X702_splice: 5/5666, Q736*: 2/2286 supporting reads), and no evidence of a mutation in P19 (Table S1). Among the cohort, five samples had sufficient leftover cfDNA libraries to repeat the hybridization capture as technical replicates. We observed concordance between the VAF in both replicates (Figure S1A) with a Pearson correlation of *r* ^2^ = 0.993.

**Figure 3:**
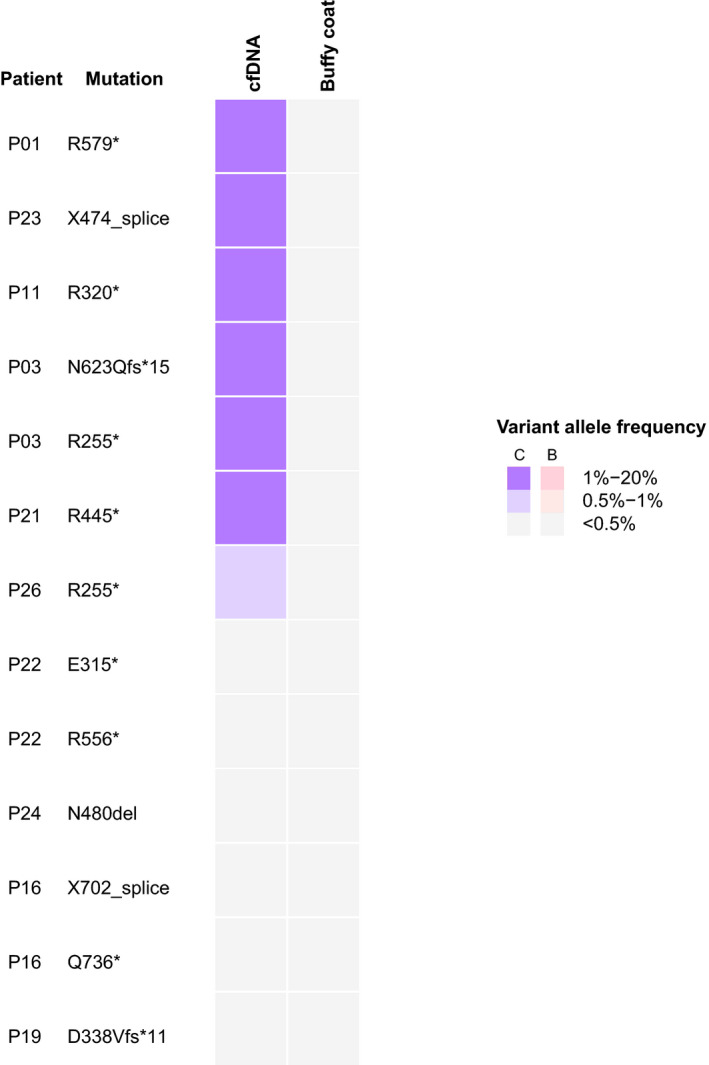
De novo identification of somatic *RB1* mutation. Results in both cfDNA and buffy coat are shown. BC, buffy coat; cf, cfDNA

In practice, tumor tissue is not always available for mutation discovery, requiring de novo identification of *RB1* mutations from plasma cfDNA without prior knowledge. To demonstrate the feasibility of this analysis, we performed de novo calling in the cfDNA samples without referring to information known from the corresponding tumors. Using the criteria outlined in the Methods, we identified *RB1* mutations in 6 of 10 patients in the cohort (7 of 13 mutations), which agreed with the known mutations from matched tumors (Figure 3). Subsequent de novo analysis on the five technical replicates mentioned above revealed concordance between each replicate (Figure S1B). In addition to the expected mutations, based on the same criteria, we also identified two additional *RB1* mutations at 1.56% and 0.99% VAF in cfDNA, respectively, that were not reported in the corresponding tumors (Table S2). We reviewed the tumor mutation data to check if these mutations were present below the detection threshold of MSK‐IMPACT, but could not find evidence of them (P22, p.G509E: 0 reads/280 reads; P26, p.P781Q: 0 reads/741 reads). We cannot rule out the possibility that these 2 mutations may be false positive, or could possibly be mosaic mutations derived from a subpopulation of cells from the eye ^34^ Unfortunately, we did not have sufficient materials to repeat the analysis to verify the possibility of false positive, or another sample from the respective patients to verify the possibility of mosaicism. The current bioinformatics analysis has yet to be improved by interrogating a panel of healthy donor plasma samples to systematically profile the error rate at the level around or below 1% VAF.

In summary, we demonstrated feasibility to detect *RB1* mutations in cfDNA of patients with advanced intraocular disease, 3/10 of whom went on to develop metastatic disease, with an ability to detect RB1 mutation in 8/10 patients by tumor‐guided genotyping, and in 6/10 patients without tumor information by de novo mutation identification.

## 4 DISCUSSION

This proof of concept study demonstrates that plasma cfDNA analysis has the potential to detect somatic *RB1* mutations in patients with advanced unilateral retinoblastoma without a detectable germline mutation. We applied two different approaches: genotyping known *RB1* mutations guided by the matched tumor, and de novo analysis without referring to knowledge from the tumor. The former is more sensitive, and the latter is more applicable in the situation where tumor may not be available. In both scenarios we were able to detect somatic *RB1* mutations in plasma of a majority of the patients.

Our results revealed several interesting observations. It was observed that plasma analysis can potentially detect somatic *RB1* mutations that are present as the second hit in addition to the germline *RB1* mutation in a given patient. For example, patient P01 was reported to carry an *RB1* germline mutation according to a separate clinical test. Interestingly, tumor and cfDNA analysis identified a different *RB1* mutation, present at VAF of 1.37% in cfDNA and not detectable in the matched buffy coat, suggesting that it is likely somatic, as germline variants detected in cfDNA are typically at a VAF around 50%. This patient went on to develop bilateral disease. This somatic mutation identified in the cfDNA is likely the second hit to the *RB1* gene that are associated with the development of bilateral retinoblastoma.

The VAFs of *RB1* mutations in cfDNA tend to be higher in patients that went on to develop metastatic disease. Of the three patients that developed metastasis, two patients (P23 and P11) had the highest *RB1* mutation VAFs from cfDNA in this study (12.6% and 8.1% respectively). P23 had metastatic recurrence of disease 3 months after eye enucleation with right orbit recurrence and bone marrow disease, whereas other patients in this cohort who went on to develop metastatic disease did so at an average of 10 months from initial diagnosis and enucleation (P11, P16). It is likely the higher VAF noted in P23’s sample is representing a higher disease burden^27^ or microscopic metastatic disease. In the other two patients (P11 and P16) who eventually developed metastasis, the cfDNA sample from P11 showed the second highest VAF at 8.1%; however, both mutations in P16 were only identified at subthreshold levels. Although higher VAFs may correspond with a higher risk of metastatic disease, a larger sample cohort would be required to truly determine the correlation. These findings also suggest that longitudinally tracking *RB1* mutations in cfDNA may allow for early detection of disease recurrence. Indeed, feasibility has been demonstrated in adult solid tumors.^35^, ^36^ Further work needs to be done to understand the implication of changes around low‐level VAF (eg <1%), as variations may be introduced by sampling error and should not be mistaken as tumor response. Nonetheless, our results agree with the positive correlation between disease metastasis and tumor cfDNA levels reported in other solid tumors.

The level of tumor‐derived cfDNA in plasma likely drops after systemic chemotherapy, thus hindering the ability to detect the *RB1* mutations in cfDNA. For example, in the 7 patients with unilateral retinoblastoma, we detected the *RB1* mutations from cfDNA in 6 of 7 samples, except sample P19 despite a unique coverage of 1596x. The mutation may be present in the plasma at levels below the theoretical detection limit of 3 in 1596 (0.18%). P19 is the only patient in the cohort that received 1 cycle of systemic chemotherapy prior to enucleation (due to the family's preference for eye salvage). It is possible that the amount of circulating tumor‐derived cfDNA had already decreased at the time of blood draw as a result of this therapy.^37^ Therefore, the timing of blood draw is critical if the intent is to determine somatic *RB1* status for diagnostic purposes.

To apply this tool in the clinic for definitive identification of somatic *RB1* mutations, it is critical to validate the analytical performance of the assay to ensure the accuracy, reproducibility, limits of detection, and false positive rate are fully characterized. When performing de novo mutation identification in cfDNA, it will be important to further evaluate the false positive rate by analyzing a large panel of healthy donor's plasma cfDNA. The sensitivity and accuracy of detecting low VAF *RB1* mutations could theoretically be improved by analyzing the samples in replicates, or by incorporating unique molecular identifiers with deeper sequencing to reduce the background errors introduced during the process.^38^, ^39^ High confident mutation discovery below 1% VAF will also be important for identifying mosaic *RB1* mutations derived from sub‐population of the eyes that are absent in the tumor. The identification of low level mosaicism would be an interesting to explore in future study by collecting multiple sample types from the same patient across time to observe if the mutation levels persist or change with treatment.

It is worth noting that the patients in this study had advanced intraocular disease with a relatively higher rate of metastasis than the general retinoblastoma population. This is partly because most of the retinoblastoma patients at MSKCC received treatment that preserved their vision, and in this study we deliberately included patients who have tumor samples available as a result of eye enucleation such that we can compare the *RB1* mutation status in plasma with tumor. The stage of disease seen in these patients, although advanced, was still limited to a unilateral eye at the time of diagnosis. As it is known that advanced tumors shed more tumor derived cfDNA^27^, ^37^; future studies are needed to determine the feasibility to detect somatic *RB1* mutations from cfDNA in patients with earlier stages of retinoblastoma. Besides *RB1* mutations, a small proportion of patients (1%) with retinoblastoma may have *MYCN* amplifications driving their disease, not an *RB1* loss of function mutation.^10^, ^40^, ^41^ Although the current study focuses on *RB1* mutation analysis, previous work has shown that cfDNA can also reveal tumor‐derived somatic copy number alterations including changes in *MYCN*.^42^, ^43^, ^44^ Incorporating mutational and copy number analysis can potentially expand the scope of patient population and the sensitivity of detecting tumor‐derived cfDNA at diagnosis and in the relapse setting. Blood samples are safe to obtain and could potentially eliminate the risks of repeated exams under anesthesia in such young patients. Germline mutation analysis could also be completed using the same methodology by looking for the *RB1* mutation in both the buffy coat DNA and plasma cfDNA from a single blood sample which would be useful for patients with both unilateral and bilateral disease presentation.

In conclusion, this study demonstrates the feasibility of using plasma cfDNA to obtain a molecular profile of retinoblastoma by determining the key mutations in the *RB1* gene. As treatment of retinoblastoma continues to improve ocular survival, fewer tissue samples will be available to determine the tumor's mutational profile, and an alternative method to determine the *RB1* mutation status will be required. Identification of an *RB1* mutation by cfDNA and demonstration of its absence in the buffy coat can potentially determine whether or not the patient has a somatic *RB1* mutation. Although examination under anesthesia cannot be fully eliminated, *RB1* gene monitoring via plasma cfDNA has the potential to provide data to supplement the clinical management in these young patients. This will minimize their exposure to sedation and the risks associated with each examination. The results from this proof of concept study should be further evaluated in large cohorts that involve all stages of retinoblastoma patients, using a fully validated assay to further improve the accuracy of detecting low‐level *RB1* mutation in plasma DNA. We envision that plasma cfDNA analysis will play an important role in both diagnosis and monitoring of response to treatment in patients with retinoblastoma.

## DATA AVAILABILITY STATEMENTS

The mutation data are available in the cBioPortal for Cancer Genomics at the following URL: https://www.cbioportal.org/study/summary?id=rbl_cfdna_msk_2020

## Conflict of Interest

PK, JY, CMS, DS, XJ, JE, KH, AV, JHF, NS, and DHA have nothing to disclose. MFB, DWYT, JP, MH, and FM are co‐inventors on a provisional patent application for systems and methods for detecting cancer via cfDNA screening (PCT/US2019/027487). DT and FM are co‐inventors on a provisional patent application for systems and methods for distinguishing pathological mutations from clonal hematopoietic mutations in plasma cell‐free DNA by fragment size analysis. MFB has received consulting fees from Roche and grant support from Illumina and Grail. DT has received research support from ThermoFisher Scientific, EPIC Sciences, speaking honoraria and travel support from Nanodigmbio, Cowen, BoA Merrill Lynch. IJD reports non‐financial support from Apexigen, personal fees from Bayer, grants from Bristol‐Myers Squibb, personal fees from Celgene, grants from Genentech, grants from Novartis, outside the submitted work.

## Author Contributions

PK, NS, IJD, and DWYT contributed to conceptualization. JE, JHF, DHA, and IJD contributed to patient recruitment and sample collection. PK, FM, XJ, KH, AV, and MFB contributed to collection and assembly of data. Data Curation and Formal Analysis: PK, JY, FM, CMS, DS, MH, JP, MFB, IJD, and DWYT contributed to data curation and formal analysis. PK wrote the first draft of the paper and all authors approved the final version.

## Supporting information

Fig S1

Table S1
