# Supplementary material for: Cell‐free DNA profiling in retinoblastoma patients with advanced intraocular disease: An MSKCC experience
Source: Cancer Med. 2020 Jul 7;9(17):6093–101. doi: 10.1002/cam4.3144 (PMC7476838; doi:10.1002/cam4.3144)

Supplementary Figure 1: Variant allele frequencies (VAF) noted in technical replicates from P01, P03, P11, P16 and P19. Figure 1A: Technical replicates analyzed by Waltz genotyper are shown here. Good concordance was noted between the replicates; pearson correlation $r^2 = 0.993$. Figure 1B: Technical replicates analyzed by Vardict for *de novo* calls are shown below. Pearson correlation between replicates, $r^2 = 0.997$.

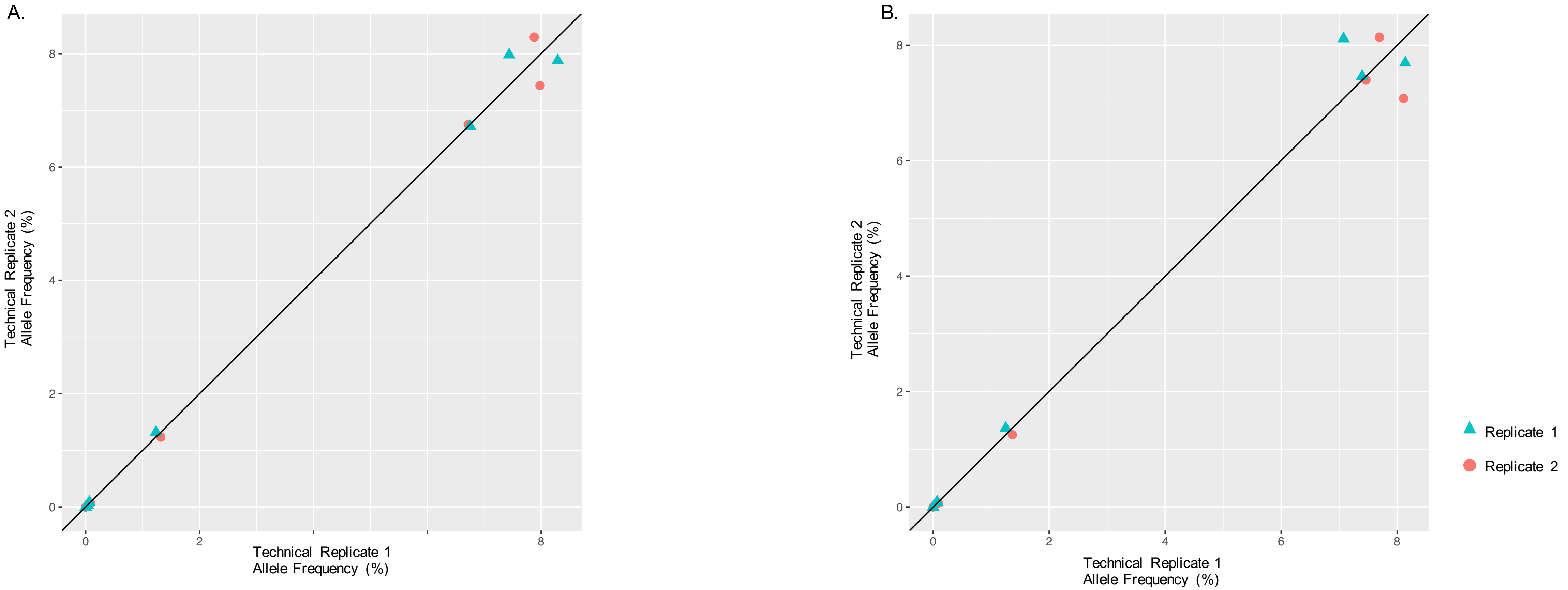

Supplement: Supplementary file 1 — Fig S1 [file CAM4-9-6093-s001.pdf]
